# Prediction of OncotypeDX recurrence score using hematoxylin and eosin-stained whole slide images

**DOI:** 10.1038/s41523-026-00937-w

**Published:** 2026-05-11

**Authors:** Shachar Cohen, Gil Shamai, Edmond Sabo, Alexandra Cretu, Iris Barshack, Tal Goldman, Gil Bar-Sela, Alexander T. Pearson, Dezheng Huo, Frederick M. Howard, Ron Kimmel, Chen Mayer

**Affiliations:** 1 Taub Faculty of Computer Science, Technion-Israel Institute of Technology, Haifa, Israel; 2 Department of Pathology, Carmel Medical Center, Haifa, Israel; 3 Ruth and Bruce Rappaport Faculty of Medicine, Technion-Israel Institute of Technology, Haifa, Israel; 4 Department of Pathology, Sheba Medical Center, Tel Hashomer, Ramat-Gan, Israel; 5 Department of Pathology, Faculty of Medicine, Tel Aviv University, Tel Aviv, Israel; 6 Department of Pathology, Emek Medical Center, Afula, Israel; 7 Department of Oncology, Emek Medical Center, Afula, Israel; 8 Technion Integrated Cancer Center, Faculty of Medicine, Technion-Israel Institute of Technology, Haifa, Israel; 9 Department of Medicine, University of Chicago, Chicago, IL USA; 10 Department of Public Health Sciences, University of Chicago, Chicago, IL USA; 11 Faculty of Electrical and Computer Engineering, Technion-Israel Institute of Technology, Haifa, Israel

**Keywords:** Biomarkers, Cancer, Computational biology and bioinformatics, Oncology

## Abstract

The OncotypeDX 21-gene assay guides adjuvant chemotherapy decisions in early-stage, hormone receptor–positive, HER2-negative breast cancer, but cost and turnaround time limit access. This study presents a deep learning-based approach for predicting OncotypeDX recurrence scores directly from hematoxylin and eosin-stained whole slide images. Our approach leverages a deep learning foundation model pre-trained on 171,189 slides via self-supervised learning, which is fine-tuned for our task. The model was developed and validated using five independent cohorts, out of which three are external. On the two external cohorts that include OncotypeDX scores, the model achieved an AUC of 0.836 and 0.817, and identified 22% and 16.3% of the patients as low-risk with sensitivity of 0.97 and 0.97 and negative predictive value of 0.97 and 0.96, showing strong generalizability despite variations in staining protocols and imaging devices. Kaplan-Meier analysis demonstrated that patients classified as low-risk by the model had a significantly better prognosis than those classified as high-risk, with a hazard ratio of 4.1 (*P* < 0.001) and 2.0 (*P* < 0.01) on the two external cohorts that include patient outcomes. This artificial intelligence-driven solution offers a rapid, cost-effective, and scalable alternative to genomic testing, with the potential to enhance personalized treatment planning, especially in resource-constrained settings.

## Introduction

Breast cancer remains one of the most prevalent malignancies worldwide, with an estimated 2.3 million new cases diagnosed annually^1^. The management of breast cancer has evolved considerably in recent years, shifting towards more personalized treatment approaches. Central to this paradigm shift is the use of genomic assays, which provide crucial information about tumor biology and help guide treatment decisions, particularly regarding the necessity of adjuvant chemotherapy. OncotypeDX, a 21-gene expression assay, has become a standard tool in clinical practice for early-stage, hormone receptor-positive, human epidermal growth factor receptor 2 (HER2) negative breast cancers^2^. Utilizing RT-PCR gene expression profiling, this test generates a recurrence score (RS) that estimates the risk of distant recurrence. Patients classified as high-risk (RS ≥ 26) were shown to benefit from chemotherapy, whereas no chemotherapy benefit was observed for low-risk (RS < 11) and intermediate-risk (11 ≤ RS < 26) patients^3,4^. While OncotypeDX offers substantial clinical benefits, it is associated with significant costs and turnaround time, which can delay treatment initiation and limit accessibility in resource-constrained settings. In contrast, AI-based alternatives also require digital pathology infrastructure (slide digitization and computation), which entails upfront and maintenance costs, but these can be amortized across many cases and applications.

In recent years, the field of digital pathology has witnessed remarkable advancements, particularly in the application of artificial intelligence (AI) to hematoxylin and eosin (H&E)-stained whole-slide images (WSI)^5–10^. These developments have opened new avenues for extracting valuable molecular and prognostic information from routine histopathological slides^11–22^.

Several previous studies have explored AI-based methods for predicting OncotypeDX scores, often combining histopathology with clinical or textual data^23–26^. However, these approaches have generally shown low generalizability to external cohorts and included relatively few or small-sized external datasets, highlighting the need for robust models that generalize well across diverse clinical environments.

Self-supervised learning (SSL) enables the extraction of meaningful features from large amounts of unlabeled data, which is especially valuable in pathology, where labeled data is limited and expensive to obtain. Foundation models, trained using SSL, make use of especially large and diverse datasets across institutions, improving their ability to generalize. This is particularly important in clinical practice, where variability in slide preparation, staining, and imaging protocols can affect performance.

In this study, we aim to develop a robust, foundation-model-based system for predicting the OncotypeDX RS directly from H&E-stained WSI. By leveraging a foundation model—trained via SSL on a large number of slides—we aim to create a system capable of generalizing across diverse clinical environments despite variations in staining and imaging protocols. Furthermore, we utilize a multi-institutional dataset spanning five independent cohorts to validate the system’s ability to provide immediate and accurate risk stratification in real-world settings. Our objective is to determine if this approach can help make precision oncology more accessible, particularly where genomic testing is unavailable.

## Results

### Cohort clinico-pathological characteristics

Clinical and pathological features are summarized in Supplementary Table 1. The combined cohort included 4,227 patients from Israel (Carmel, Haemek, Sheba), the United States (UCMC), and Australia (ABCTB). Among cohorts with available OncotypeDX data, high-risk prevalence (RS ≥ 26) ranged from 14.5% (Carmel) to 20.6% (Sheba), with mean RS ranging from 16.14 to 19.37. Median age ranged from 56 to 67 years, and mean tumor size (where available) ranged from 1.87 cm (Sheba) to 2.61 cm (ABCTB). Grade 2 tumors predominated across cohorts, ER positivity exceeded 98%, and PR positivity ranged from 81.6 to 89.5%. Ki67 was available for Carmel, Haemek, and Sheba (means 15.81–18.30%), while HER2 was available only for Sheba (mean 0.7). These baseline characteristics confirm the clinical heterogeneity across the cohorts, which is critical for evaluating model generalizability.

### Model evaluation on the internal and external test sets

**Fig. 1:**
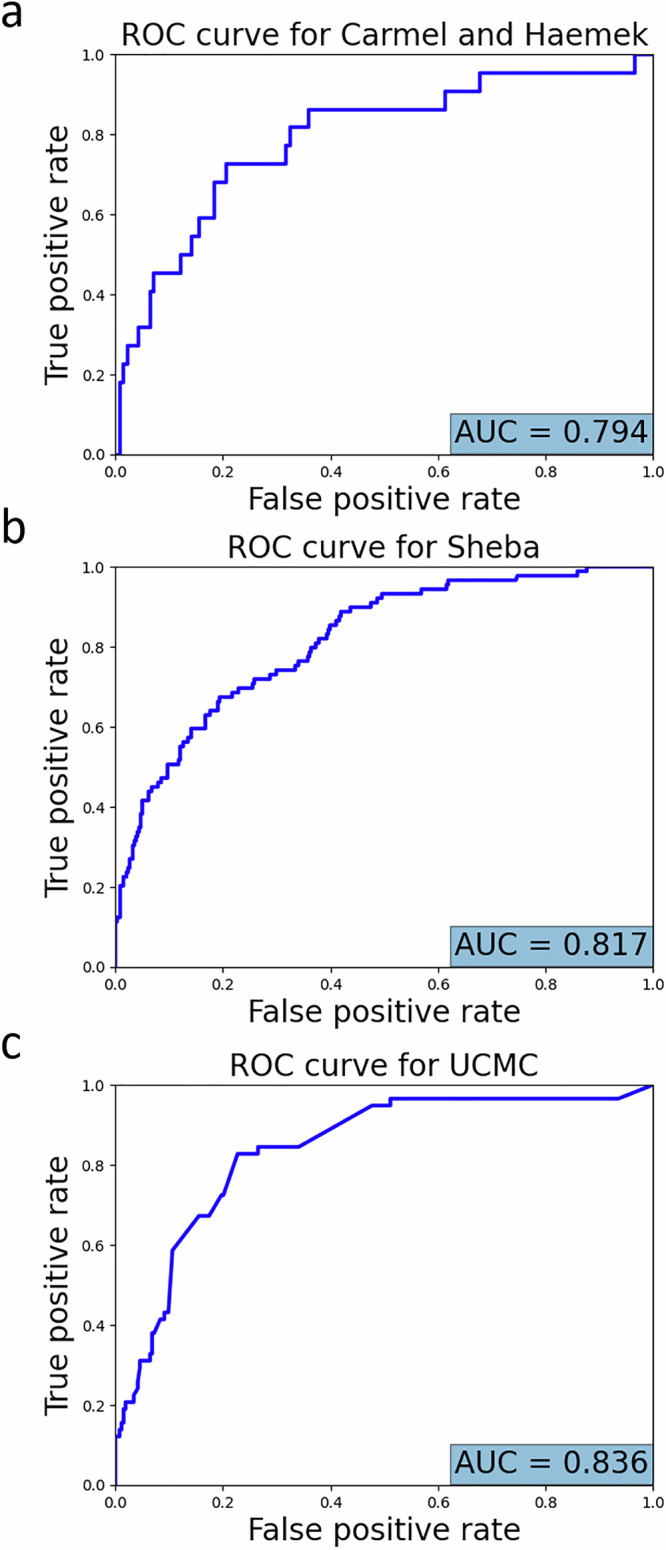
Model AUC performance. ROC curves obtained by the model for predicting high genomic risk (RS ≥ 26) for: **a** the test set of the internal cohorts (Carmel and Haemek). **b** Sheba. **c** UCMC. AUC value for each graph is specified at the bottom.

We trained and validated our model using the training set and test set of the combined internal cohorts (Carmel and Haemek). The model achieved an AUC of 0.794 (95% CI: 0.672–0.897) for predicting high OncotypeDX risk (RS ≥ 26) on the combined internal test set (Fig. 1a and Table 1). This shows that the model is capable of separating patients into low and high genomic risk groups with high reliability. Next, we validated the model on the Sheba and UCMC external cohorts, which had OncotypeDX RS data. The model achieved an AUC of 0.817 (95% CI: 0.77–0.868) on the Sheba cohort (Fig. 1b) and an AUC of 0.836 (95% CI: 0.774–0.889) on the UCMC cohort (Fig. 1c), indicating that the model generalizes well to external cohorts. The model achieved a Pearson correlation coefficient of 0.605 and a Spearman correlation coefficient of 0.547 on the Sheba cohort. On the UCMC cohort, the model achieved a Pearson correlation coefficient of 0.519 and a Spearman correlation coefficient of 0.481.

**Table 1:** Model AUC performance across the datasets for predicting high genomic risk

Dataset:	Carmel validation	Carmel test	Haemek validation	Haemek test	Sheba	UCMC
AUC per patient (95% CI):	0.811 (0.742–0.875)	0.779 (0.642–0.898)	0.843 (0.755-0.921)	0.834 (0.6189–1)	0.817 (0.77–0.868)	0.836 (0.774–0.889)
AUC per slide (95% CI):	0.82 (0.772–0.863)	0.807 (0.717–0.881)	0.859 (0.782–0.922)	0.856 (0.649–1)	0.783 (0.736–0.821)	0.826 (0.788–0.871

AUC performance for predicting high genomic risk (RS ≥ 26) at the patient and slide level for each of the datasets. 95% CIs are indicated.

### Performance evaluation in clinico-pathological subgroups

Model discrimination remained relatively consistent across clinically relevant subgroups (Supplementary Fig. 1). For grade 2 tumors, AUCs were 0.749 (95% CI: 0.569–0.902) (Carmel and Haemek), 0.779 (95% CI: 0.696–0.852) (Sheba), and 0.749 (95% CI: 0.657–0.832) (UCMC). For grade 3 tumors, AUCs were 0.825 (95% CI: 0.577–0.975) (Carmel and Haemek), 0.775 (95% CI: 0.671–0.86) (Sheba), and 0.750 (95% CI: 0.639–0.854) (UCMC). Using a Ki67 cutoff of 20%, AUCs in Carmel and Haemek were 0.851 (95% CI: 0.713–0.964) (Ki67 low) and 0.772 (95% CI: 0.546–0.946) (Ki67 high), while in Sheba they were 0.713 (95% CI: 0.555–0.863) (Ki67 low) and 0.806 (95% CI: 0.738–0.868) (Ki67 high). Performance for grade 1 tumors is not reported because high-risk events (RS ≥ 26) were too rare to support a reliable AUC estimate (UCMC: 7 grade 1 high-risk cases; Carmel/Haemek/Sheba: 0). Invasive lobular carcinoma (ILC) performance was assessed using correlation due to the limited number of high-risk (RS ≥ 26) ILC cases. In Sheba, ILC patients (*n* = 23) showed Pearson *r* = 0.425 and Spearman r = 0.511 between the model-predicted score and ground-truth RS, whereas in UCMC, the correlation was lower (ILC *n* = 71; Pearson *r* = 0.125, Spearman *r* = 0.310). The reduced correlation in ILC may reflect both the lower prevalence of ILC in the data (and thus fewer subtype-specific examples for learning) and the possibility that RS-associated histologic patterns are less pronounced or different in ILC.

### Evaluation of discrimination ability versus cut-off threshold

The initial validation studies, NSABP B-20 and SWOG-8814, demonstrated significant chemotherapy benefit in patients with RS ≥ 31^2,27^. A reanalysis of the B-20 cohort indicated that RS ≥ 26 provided a more appropriate threshold for the prediction of chemotherapy benefit^28,29^, which was later used in the TAILORx study^3^. Yet, the optimal OncotypeDX threshold to determine chemotherapy benefit remains uncertain^30^. We next evaluated the model’s ability to discriminate low and high-genomic risk patients for cut-off thresholds other than 26. Namely, for each threshold ***t***, we computed the AUC and balanced AUPRC performance of the model for predicting OncotypeDX RS≥***t*** in the internal and external cohorts (Supplementary Fig. 2). The analysis shows that the AUC and balanced AUPRC performance steadily rose with respect to ***t***, indicating that the model’s ability to distinguish between low and high genomic risk improved with higher cut-off thresholds. Both AUC and balanced AUPRC were computed to evaluate both the actual model performance and the effects of potential imbalances between the positive and negative classes on model performance. When computing AUPRC, we chose to balance the data through subsampling the majority class to ensure a fair comparison of the performance for different thresholds. Interestingly, this indicates that cut-off thresholds higher than 26 exhibited more distinctive morphological characteristics.

### Evaluating the model’s clinical utility

**Fig. 2:**
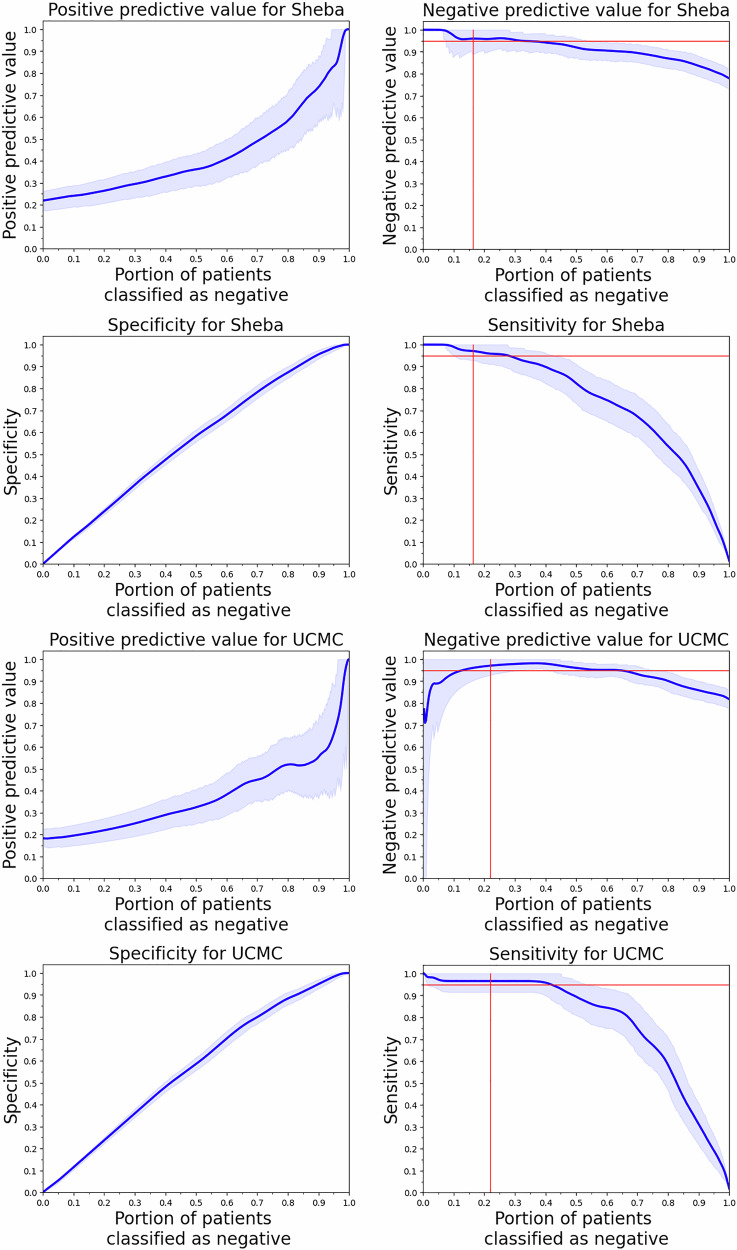
Clinical utility evaluation on the external cohorts. PPV, NPV, specificity, and sensitivity per portion of patients classified as negative for the external test sets (Sheba and UCMC) (dark blue line) and 95% CI (light blue interval). Red lines extended at the low-risk cutoff threshold for each cohort and the 95% NPV and sensitivity points.

We next aimed to assess the clinical utility of the model. To this end, we evaluated the model’s specificity, sensitivity, NPV, and PPV on the Sheba and UCMC cohorts for different cut-off thresholds (Fig. 2). Notably, when using a high sensitivity threshold, computed in a manner used in previous studies^31^ to achieve sensitivity = 0.95 on the internal cohorts, the model could identify 22% and 16.3% of the patients who had a low genomic score, with sensitivity of 0.97 and 0.97 and NPV of 0.97 and 0.96, showing strong performance in identifying low genomic risk patients (OncotypeDX < 26). This demonstrates the model’s potential to spare unnecessary chemotherapy for many patients, which may be particularly relevant in countries without access to genomic assays.

### Incorporation of clinical data

**Fig. 3:**
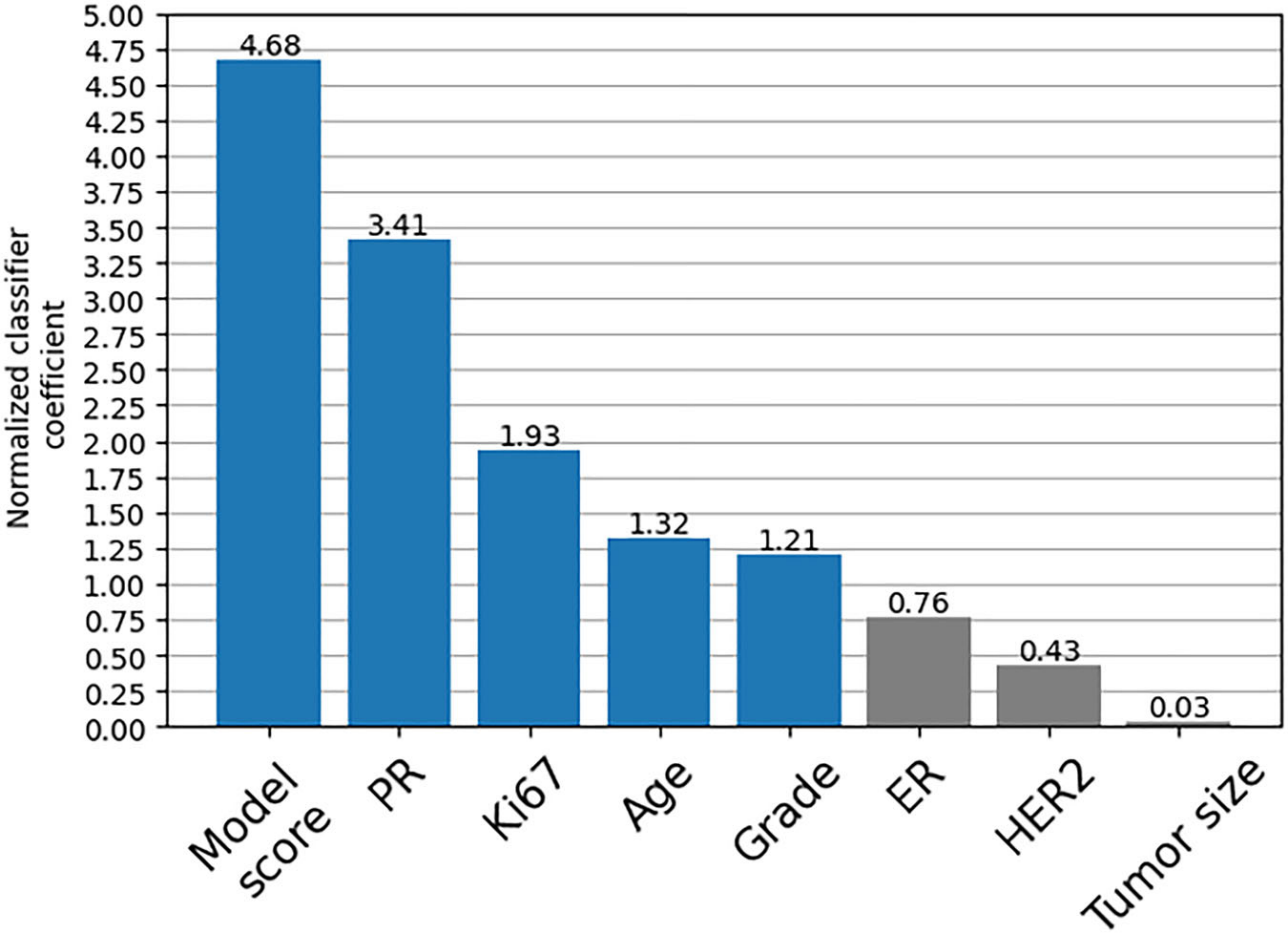
Feature importance by coefficient score. Feature importance is shown by normalized coefficient values for the linear classifier trained on clinical features and slide model scores in the Sheba cohort. The exact normalized coefficient (absolute value) is displayed above each bar, with statistically significant features in blue and non-significant features in grey.

We next evaluated the potential benefit of including clinical variables in the training process. To this end, we trained a linear classifier to predict the OncotypeDX scores from both H&E and clinical variables on the Sheba cohort, which had sufficient clinical variables available (Methods). We then conducted a feature importance analysis, which revealed that the model-predicted score was the most significant predictor, followed by the PR expression as the second most important feature. Interestingly, the model-predicted score, PR expression, Ki67 expression, age, and tumor grade were found to be statistically significant (*P* < 0.05), while ER expression, HER2 class, and tumor size were not (Fig. 3).

### Survival analysis

**Fig. 4:**
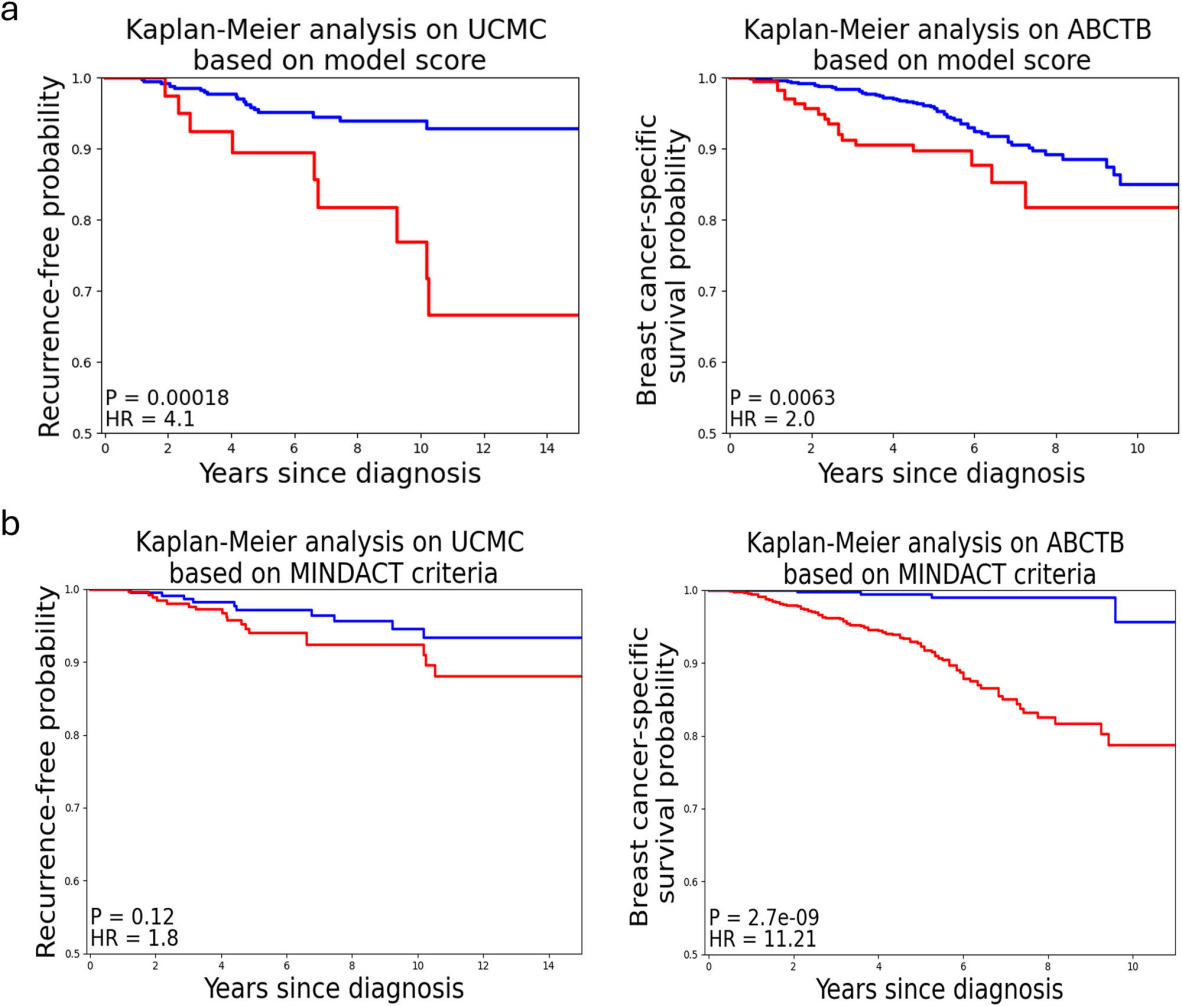
Survival analysis for UCMC and ABCTB based on model score and MINDACT clinical risk. Kaplan-Meier analysis on UCMC and ABCTB based on: **a** calibrated model score with the high-risk cutoff model score ≥ 26. **b** MINDACT clinical risk criteria. A blue curve represents patients classified as low-risk, and patients classified as high-risk are represented by a red curve. The portion of patients in each risk group, as well as *P* values and HR, are indicated at the bottom.

We next evaluated the model’s ability to estimate patient prognosis. We stratified the patients in the ABCTB and UCMC cohorts into low and high-risk groups based on the calibrated model scores (Fig. 4a). On UCMC, our endpoint was recurrence-free interval, achieving HR = 4.1 (*P* < 0.001). On ABCTB, our endpoint was breast cancer-specific survival because no recurrence data were available in this cohort, achieving HR = 2.0 (*P* < 0.01). For comparison, we also stratified patients using the MINDACT clinical risk criteria^32^ (Fig. 4b), achieving HR = 1.8 (*P* = 0.12) on UCMC and HR = 11.21 (*P* < 1e-08) on ABCTB. Notably, MINDACT clinical risk showed strong prognostic separation for ABCTB breast cancer-specific survival but was not significant for UCMC recurrence-free interval.

### Unsupervised feature clustering and visualization

**Fig. 5:**
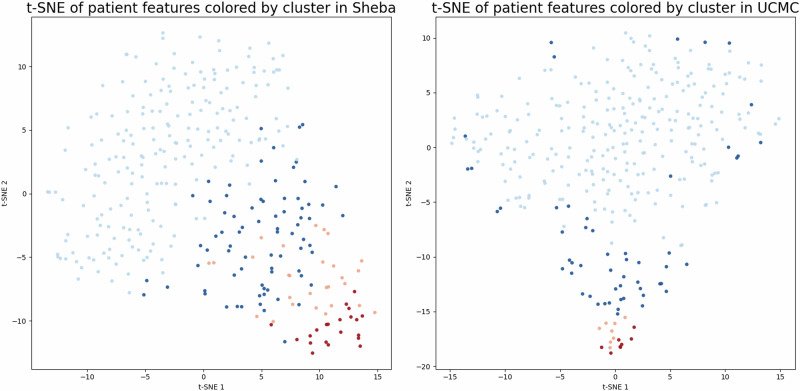
t-SNE visualization of unsupervised clusters in the learned patient embedding space (Sheba and UCMC). Patient-level feature embeddings were extracted from the model and clustered using k-means (*k* = 2) separately within the model score < 26 and model score ≥ 26 groups, yielding four clusters per cohort. Embeddings were projected to two dimensions using t-SNE and colored by cluster assignment. Mean ground-truth OncotypeDX RS for each cluster is reported in the legend. Clustering was performed without using RS labels; RS values are shown only for post hoc cluster characterization.

To examine whether the learned embeddings exhibit structure consistent with genomic risk, we applied the unsupervised clustering and visualization analysis described above (Fig. 5). Despite using no RS labels, the resulting clusters showed an ordered relationship with ground-truth RS. In Sheba, the two clusters within the model score <26 group had mean RS = 16.50 and 20.58, whereas the two clusters within the model score ≥26 group had mean RS = 29.77 and 36.26. In UCMC, mean RS increased from 16.27 and 23.46 in the model score <26 clusters to 33.20 and 33.45 in the model score ≥26 clusters. In the t-SNE maps, the more extreme low-risk and high-risk clusters were clearly separated, while clusters nearer the decision boundary showed greater proximity, consistent with borderline cases.

## Discussion

In this study, we demonstrated that our AI-driven model can reliably predict OncotypeDX scores and estimate patient prognosis directly from routine H&E-stained tissue slides, suggesting that it is able to capture critical histopathological features linked to tumor biology that influence recurrence risk. Histologically, H&E-stained slides provide a wealth of information about the structural and cellular composition of tumors, including features such as nuclear atypia, mitotic activity, stromal characteristics, and the extent of immune infiltration. These features are known to reflect underlying biological processes, such as proliferation, genetic instability, and tumor microenvironment interactions, which are directly linked to recurrence risk and response to therapy. While traditional pathology relies on human interpretation to identify these features, AI excels at detecting subtle, complex patterns and relationships within the data that may not be readily apparent to the human eye.

A key innovation in our approach is the use of a foundation model—Prov-GigaPath— which was pretrained using SSL. This deep learning model is pre-trained on a large and diverse pathology dataset and fine-tuned for the specific task of predicting OncotypeDX scores from H&E-stained slides. Foundation models have demonstrated effectiveness across various domains, including natural language processing, computer vision, and medical imaging, due to their ability to learn transferable representations from broad, unlabeled data^33–35^. This approach is particularly advantageous in clinical contexts, where curated annotations are costly, class distributions are imbalanced, and privacy constraints limit data sharing.

By starting from a broadly pre-trained model, we were able to reduce reliance on task-specific data while achieving strong predictive performance. Crucially, the model’s pretraining on heterogeneous pathology data enhances its ability to generalize across institutional, demographic, and technical variability, such as differences in staining protocols or imaging devices, supporting its robustness in real-world applications. This generalizability is a key benefit of the foundation model paradigm and underpins its suitability for scalable clinical deployment.

Previous studies have shown the potential of predicting OncotypeDX from H&E-stained slides^23,24^. However, these studies exhibited limitations in generalizability and were constrained by relatively small training datasets and narrower validation cohorts. Boehm et al. reported strong internal performance with their image-only model (AUC 0.85). However, they showed a decline in AUC on their external cohorts (0.81 and 0.80), which included 1027 patients^23^. Similarly, Goyal et al. also demonstrated a decline in performance between the internal cohort (AUC 0.91) and the external cohort (AUC 0.84), which consisted of 405 patients^24^. In contrast, our model maintained consistent AUCs across internal (0.794) and external (0.817 and 0.836) cohorts, demonstrating the robustness of the foundation model approach. Additionally, we utilized a foundation model trained on 171,189 slides, an order of magnitude larger than foundation models used in earlier works, enabling strong generalization across diverse data sources. Our study included 727 patients from two internal cohorts and 3,500 patients from three external cohorts, encompassing substantial variation in geographic origin, patient demographics, and slide preparation protocols across multiple laboratories. These findings underscore the broad applicability of our approach, particularly in heterogeneous real-world settings.

Although our approach can reduce reliance on costly and slow genomic assays, it still requires a digital pathology workflow (slide scanning, storage, and computation), which carries non-trivial upfront and maintenance costs. However, guidance on AI-enabled digital pathology adoption in low-resource organizations emphasizes staged deployment, shared/central scanning capacity, and open-source tooling to reduce barriers^36^. In practice, reports from developing settings support feasibility. For example, a Pakistan study applied deep learning to 10× microscope-camera images uploaded into open-source QuPath, explicitly highlighting such approaches as workable in resource-limited settings and as a bridge when whole-slide scanners are unavailable^37^. Because scanner/compute investments can be amortized across high specimen volumes and multiple pathology applications, they can compare favorably to recurring per-patient genomic testing costs.

Despite the promising results, several limitations of this study must be considered. First, when we tested the inclusion of additional clinical features on the Sheba cohort, we observed that other features also held importance for prediction, and prior work suggests multimodal models can further improve risk stratification^23,38^. In line with this, our survival comparison showed that MINDACT clinical risk provided markedly stronger prognostic separation in ABCTB (HR = 11.21, *P* < 1e-08) than the image-only stratification, likely reflecting the added value of key clinical variables (e.g., nodal status), whereas MINDACT did not significantly stratify UCMC (*P* = 0.12). These findings motivate future studies that integrate our histology-derived score with a small set of routinely available clinical features to improve robustness and prognostic performance across cohorts.

Another limitation is model interpretability. While deep learning models can achieve high predictive accuracy, their decision-making processes are often opaque, which can hinder clinical adoption. To partially address this, we performed an unsupervised analysis of the learned patient embeddings: k-means clustering followed by t-SNE visualization demonstrated that clusters formed without using RS labels were nevertheless ordered by mean OncotypeDX RS (Fig. 5), suggesting that the embedding space contains structure that aligns with increasing OncotypeDX RS. However, these latent features remain difficult to map directly to specific human-interpretable morphologic attributes, and future work should focus on linking predictions to explicit histopathologic correlates using dedicated explainability approaches.

In conclusion, our work introduces a model that can reliably predict OncotypeDX scores from H&E-stained breast cancer tissue slides, offering a promising, quick, and cost-effective addition to traditional genomic assays. By enabling faster and more efficient risk stratification, this approach could significantly improve personalized treatment decisions and expand access to precision medicine for a broader range of patients.

## Methods

### Datasets used

This study utilized H&E-stained WSI of breast cancer tumors collected from five medical centers (Table 2): Carmel Medical Center (1022 slides from 569 patients), Haemek Medical Center (202 slides from 158 patients), Sheba Medical Center (697 slides from 437 patients), Australian Breast Cancer Tissue Bank (ABCTB) (3024 slides from 2552 patients) and the University of Chicago Medical Center (UCMC) (601 slides from 511 patients). OncotypeDX scores were available for the Carmel, Haemek, Sheba, and UCMC datasets, while survival data were available for the UCMC and ABCTB cohorts. Inclusion and exclusion criteria: From all cohorts, only patients with hormone receptor-positive, HER2-negative breast cancer were included. For Carmel, Haemek, Sheba, and UCMC, only patients with OncotypeDX scores were included. Slides that had physical scan issues or less than 100 available tissue tiles (see Slide segmentation and tiling) were also excluded. Other than the exclusions described above, no further data curation was done. While the ABCTB cohorts lack OncotypeDX data, all the patients included are of the same population eligible for such testing (hormone receptor-positive, HER2-negative breast cancer). As such, we utilized this cohort to evaluate the ability of our model to predict long-term clinical prognosis. Additional clinical and dataset-specific details are provided in Supplementary Table 1.

**Table 2:** Distribution of patients across the different datasets used in development and evaluation

Dataset:	Year of diagnosis	Total collected patients (slides)	Included in analysis patients (slides)	Available OncotypeDX data	Available survival data
Carmel train	2015–2022	427 (767)	425 (762)	Yes	No
Carmel test	2015–2022	142 (255)	140 (251)	Yes	No
Haemek train	2014–2022	135 (176)	133 (173)	Yes	No
Haemek test	2014–2022	23 (26)	23 (26)	Yes	No
Sheba	2014–2020	437 (697)	427 (676)	Yes	No
UCMC	2006–2020	511 (601)	490 (568)	Yes	Yes
ABCTB	2006–2015	2552 (3024)	1762 (2080)	No	Yes
Total		4227 (5546)	3400 (4536)		

Summary of the five cohorts used for development and validation in the study, three of which are from Israel (Carmel, Haemek, Sheba), one from the United States (UCMC), and one from Australia (ABCTB). We present the number of patients and slides available, the number included in the analysis, and whether or not OncotypeDX and survival data were available.

### Slide segmentation and tiling

During preprocessing, we employed Otsu’s thresholding method^39^ to distinguish between the tissue and background regions of the WSI. Following the thresholding, we divided the tissue regions into non-overlapping tiles measuring 256 × 256 at a magnification of 0.5 microns per pixel (MPP) across all cohorts.

### The proposed model

**Fig. 6:**
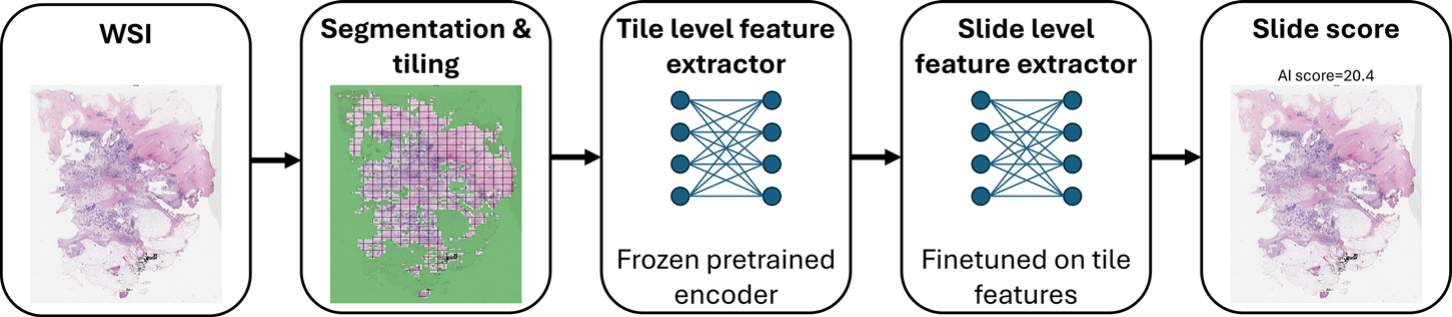
Overview of the data processing and model pipeline. The pipeline begins with tissue segmentation and tiling into 256×256 patches at 0.5 MPP. Patches are processed by a pre-trained vision transformer to extract 1,536-dimensional feature vectors. These tile embeddings are then aggregated by a fine-tuned transformer-based slide encoder to predict the final slide-level OncotypeDX recurrence score.

Our model makes use of the Prov-GigaPath foundation model, which was pre-trained on 171,189 whole-slide images^34^. Using a fixed transformer-based tile encoder, we generated a feature vector containing 1536 features per tile image. After producing a feature vector for all the tiles in a given slide, we fine-tuned a transformer-based slide encoder to predict the slide-level OncotypeDX RS. Both the slide and tile encoders were a part of the default pre-trained foundation model. During training, we used a mean squared error loss, the Adamw optimizer with an initial learning rate of 2.5e-4, and a cosine learning rate scheduler (Fig. 6).

### Model evaluation and statistical analysis

The Carmel and Haemek datasets were combined into an internal cohort used to develop the models. This internal cohort was split at the patient level, with 75% of patients assigned to the training set and 25% to the test set. The training set was further divided into 5 training folds. Slides from Sheba, ABCTB, and UCMC were used for external validation and survival analysis. Five-fold cross-validation was used to compute the predicted scores, where each model was trained on four of the five folds from the Carmel and Haemek training sets. When evaluating the model on the test sets, all five models produced a score for a given slide, and the scores were then averaged to produce a final slide score. In case of multiple slides per patient, the scores from the different slides were averaged to generate a score at the patient level. The performance metrics used for evaluation were AUC, area under the precision-recall curve (AUPRC), specificity, sensitivity, negative predictive value (NPV), and positive predictive value (PPV). The bootstrap technique with 1000 resampling iterations was used for all metrics to generate 95% confidence intervals (CIs) at the patient level. For survival analysis, Kaplan-Meier estimation was performed, and accuracy was evaluated by measuring a hazard ratio (HR) using Cox regression and *P-*value using the log-rank test. To provide a low-cost clinicopathological comparator, we also stratified patients using the MINDACT clinical risk criteria^32^ in the UCMC and ABCTB cohorts. We then performed Kaplan–Meier analyses for MINDACT low- vs high-risk groups using the same endpoints as in the model-based survival analysis.

### Performance evaluation in clinico-pathological subgroups

To assess whether performance varied across specific patient subgroups, we evaluated discrimination for predicting high genomic risk (RS ≥ 26) within tumor grade subgroups and, where available, Ki67 subgroups. Ki67 was dichotomized as low (<20%) versus high (≥20%) based on the St. Gallen consensus threshold for “high” Ki67^40^. Subgroup AUCs were computed at the patient level using the same model outputs as the primary analysis, and ROC curves were generated for visualization.

### Incorporation of clinical data

To test the potential benefit of including clinical variables, we combined the model-predicted scores with clinical data. A linear classifier was trained using the predicted scores and clinical features as inputs to predict the OncotypeDX score. The clinical features included: Ki67 expression, tumor grade, tumor size in cm, age at diagnosis, ER IHC score, PR IHC score, and HER2 class. The classifier was trained using ordinary least squares, and feature importance was evaluated by examining the normalized coefficients of the classifier. This experiment was done using the Sheba cohort because it had the entire set of clinical variables.

### Score calibration with histogram matching

While the distribution of ground-truth OncotypeDX scores in the different cohorts is relatively similar, the distribution of model-predicted scores presents some variation between cohorts, which caused an issue in some analyses. For example, when using a high sensitivity cut-off threshold to discriminate between low and high-risk patients (see Evaluating the model’s clinical utility), 22% of the patients in the internal cohort would be classified as low-risk. In comparison, 18% and 0.6% of the patients would be classified as low-risk in the UCMC and Sheba cohorts, respectively. To account for this variation, we applied histogram matching between the ground-truth OncotypeDX scores and the model-predicted scores for model calibration. To calibrate for each external cohort, we randomly sampled 100 patients from this cohort and used their data to generate a mapping function. The resulting mapping function from the histogram matching process was then applied to the model’s scores. The ground-truth, as well as model-predicted score distributions, is presented in Supplementary Fig. 3. Since ABCTB does not have ground-truth OncotypeDX data, we used patients from the internal cohort, which included the Carmel and Haemek datasets. This calibration was relevant for the survival analysis Kaplan-Meier estimation, for evaluating NPV and sensitivity when stratifying patients using a fixed threshold, and for the evaluation of the importance of clinical features when generating binary predictions based on the model scores. In those analyses, the randomly selected patients used to generate the mapping function were excluded. The rest of the presented analyses, which included model performance evaluation by computing AUC, were agnostic to the calibration, as it does not affect ranking-based metrics.

### Unsupervised feature clustering and visualization

To provide an interpretable view of the learned representation space, we performed an unsupervised clustering analysis on patient-level feature embeddings for the test cohorts with available ground-truth RS (Sheba and UCMC). For each patient, we extracted the model feature vector and, when multiple slides were available, averaged features across slides to obtain a single patient embedding. Patients were first split by the model score into low (<26) and high (≥26) predicted-risk groups, and within each split we applied k-means clustering (*k* = 2), yielding four clusters in total. For visualization, embeddings were projected into two dimensions using t-distributed stochastic neighbor embedding (t-SNE)^41^, and points were colored by cluster assignment. Ground-truth RS was not used for clustering and was only used post hoc to summarize the mean RS within each cluster.

### Ethical approvals for the datasets

The ABCTB dataset, available through the Australian Breast Cancer Tissue Bank, received ethical and scientific approval according to their access policy (see Data Availability). Informed consent was obtained from all participants in the ABCTB cohort. The Institutional Review Board (IRB) overseeing the creation of the ABCTB dataset is the Sydney Local Health District Ethics Committee (Royal Prince Alfred Zone). Contributing institutions also obtained IRB approval for sample collection and data submission, with informed consent from living participants and re-consent waived for deceased individuals. Data from Carmel, Haemek, and Sheba were collected and digitized internally for this study. Each dataset’s collection followed protocols compliant with the Helsinki Declaration and relevant institutional policies, and each dataset’s access and usage were approved in advance by corresponding local ethics boards (Helsinki approvals). Specifically, data from Carmel was approved for the research by the Helsinki committee of the Lady Davis Carmel Medical Center, data from Haemek was approved for the research by the Haemek Medical Center Ethics Committee, data from Sheba was approved for the research by the Sheba Medical Center Ethics Committee, and data from UCMC was approved for the research by the University of Chicago Institutional Review Board. Informed consent was waived by the IRBs, as the study is retrospective, involves only digital, de-identified data with no way to trace it back to patients, and does not involve any invasive or physical interventions. As each dataset adheres to its own ethics approval processes, including prior IRB or Helsinki Declaration approvals, additional IRB approval at the university level was deemed unnecessary for the use of these datasets in this study.

## Supplementary information

Supplementary Table

## Data Availability

The ABCTB dataset, accessible from the Australian Breast Cancer Tissue Bank, is subject to ethical and scientific approvals as described in their access policy: https://nsw.biobanking.org/biobanks/view/7. The remaining data collected from medical centers are not available for public access due to privacy and ethical considerations, in alignment with the Helsinki agreements and institutional policies. Interested researchers may request access directly from the respective institutions: Carmel data from the Carmel Medical Center, Israel. Haemek data from the Haemek Medical Center, Israel. Sheba data from Sheba Medical Center, Israel. UCMC data from the University of Chicago Medical Center, USA.

## Code availability

The source code used in this study has been archived in Zenodo^42^ and can be accessed at the following link: https://github.com/shachar5020/TransformerWSI4OncoDXPrediction. Statistical analysis, preprocessing, tuning of the models, and inference were done using Python 3.9.18 and Pytorch library version 2.0.0. Additional Python libraries used for database management, graphical plotting, scientific calculations, and other tasks include Numpy v.1.26.4, Pandas v.2.2.2, Scipy v.1.13.1, and Openslide v.1.3.1.
