# Supplementary material for: Prediction of OncotypeDX recurrence score using hematoxylin and eosin-stained whole slide images
Source: NPJ Breast Cancer. 2026 May 11;12:92. doi: 10.1038/s41523-026-00937-w (PMC13385769; doi:10.1038/s41523-026-00937-w)
Supplement: Supplementary file 1 — Supplementary Table [file 41523_2026_937_MOESM1_ESM.pdf]

Supplementary Table 1: Clinical characteristics of the data

	Carmel	Haemek	Sheba	UCMC	ABCTB
RS mean (std)	16.14 (9.23)	18.27 (10.47)	19.37 (9.86)	18.40 (9.72)	-
High risk (26≤RS) n/total n. (%)	82/565 (14.5%)	28/156 (17.9%)	88/427 (20.6%)	75/490 (15.3%)	-
Median age in years	67	59.5	57	56	60
Tumor size in cm<br>Median<br>Mean	 -<br>-	 1.7<br>1.99	 1.6<br>1.87	 1.7<br>2.19	 2<br>2.61
Grade n/total n. (%)<br>1 (Low)<br>2 (Intermediate)<br>3 (High)	 130/510 (25.4%)<br>332/510 (65%)<br>46/510 (9%)	 34/135 (25.1%)<br>78/135 (57.7%)<br>23/135 (17%)	 18/378 (4.7%)<br>266/378 (70.3%)<br>94/378 (24.8%)	 76/490 (15.5%)<br>325/490 (66.3%)<br>89/490 (18.1%)	 373/1753 (21.2%)<br>877/1753 (50%)<br>503/1753 (28.6%)
ER positive n/total n. (%)	564/565 (99.8%)	156/156 (100%)	427/427 (100%)	489/490 (99.7%)	1726/1761 (98%)
PR positive n/total n. (%)	480/565 (84.9%)	132/153 (86.2%)	347/425 (81.6%)	439/490 (89.5%)	1562/1748 (89.3%)
Ki67 expression mean (std)	15.81 (12.49)	18.06 (14.99)	18.30 (9.32)	-	-
HER2 expression mean (std)	-	-	0.7 (0.8)	-	-

Patient characteristics for all cohorts included in the study. For categorical variables, data is shown as a number out of the total dataset and percentage. When the variable is binary, only one category is shown, and all categories are shown otherwise. Some characteristics were not available in all cohorts or for all patients in a given cohort. When specifying the total number for a given characteristic, that number represents only the patients for whom the data was available, as opposed to all patients in the cohort. Missing fields are indicated by dashes (-).

Supplementary Figure 1: Model AUC on different patient subgroups

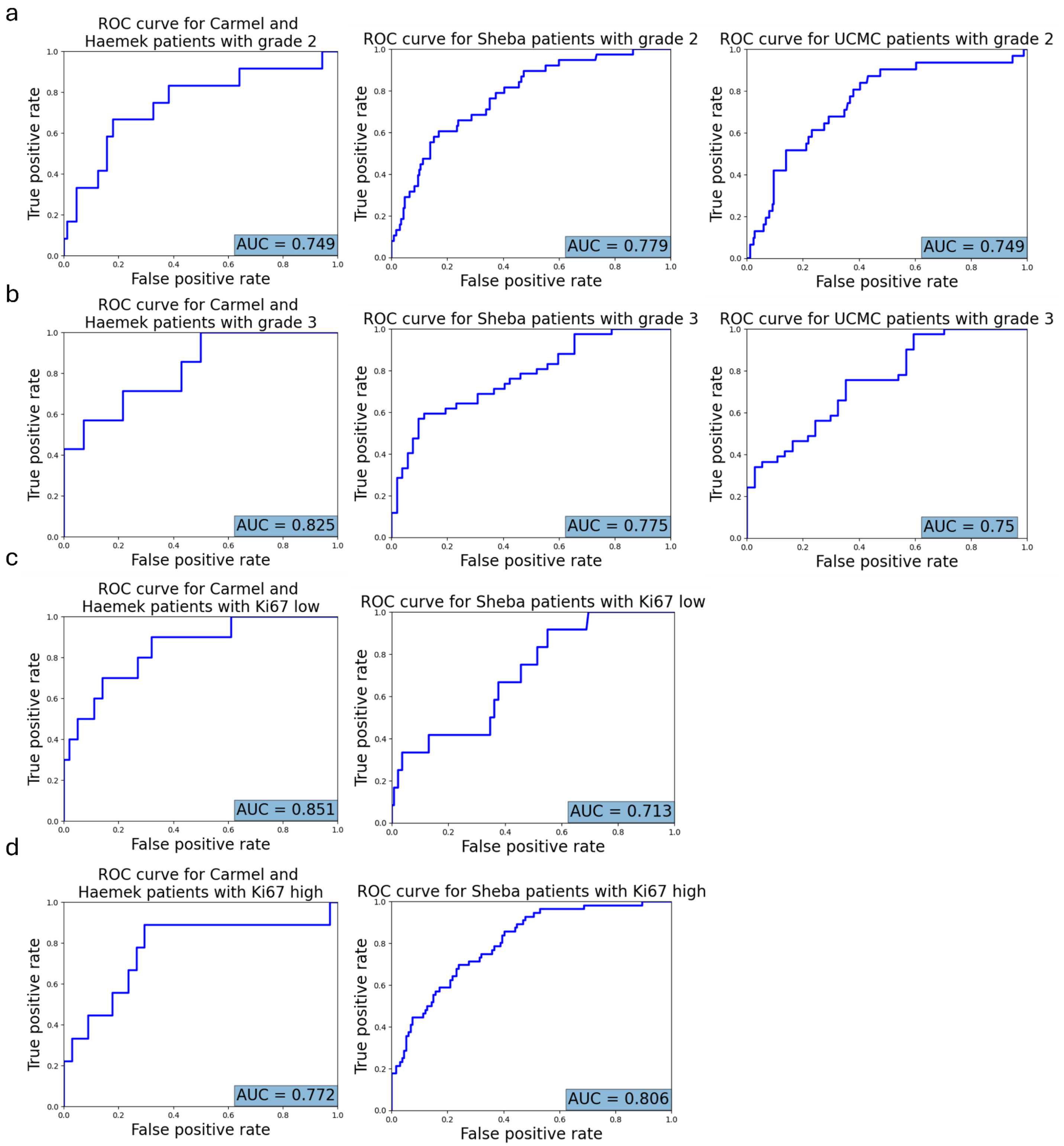

ROC curves obtained by the model for predicting high genomic risk (RS≥26) on the test set of the internal cohorts (Carmel and Haemek), Sheba, and UCMC (where possible) for: ***(a)*** *Patients with tumor grade 2.* ***(b)*** *Patients with tumor grade 3.* ***(c)*** *Patients with low Ki67 expression.* ***(d)*** *Patients with high Ki67 expression. AUC value for each graph is specified at the bottom.*

Supplementary Figure 2: Model AUC as a function of high-risk cutoff threshold

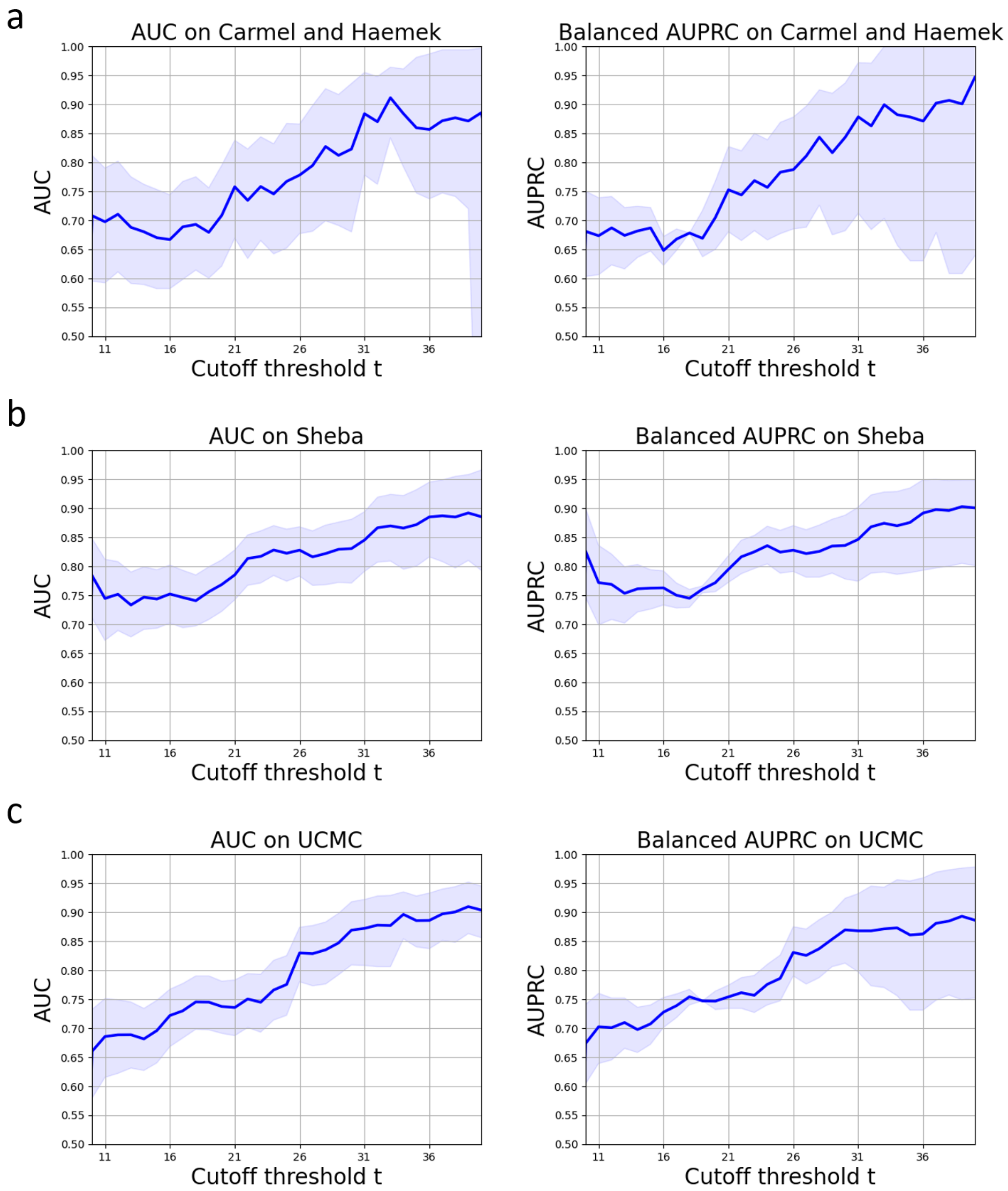

*AUC and balanced AUPRC scores obtained by the model for predicting RS≥**t**, using different cutoff thresholds **t** (dark blue line) for: **(a)** the test set of the internal cohorts (Carmel and Haemek). **(b)** Sheba. **(c)** UCMC. The 95% CIs are displayed as a light blue interval.*

Supplementary Figure 3: Violin plots representing the RS distribution in the different cohorts

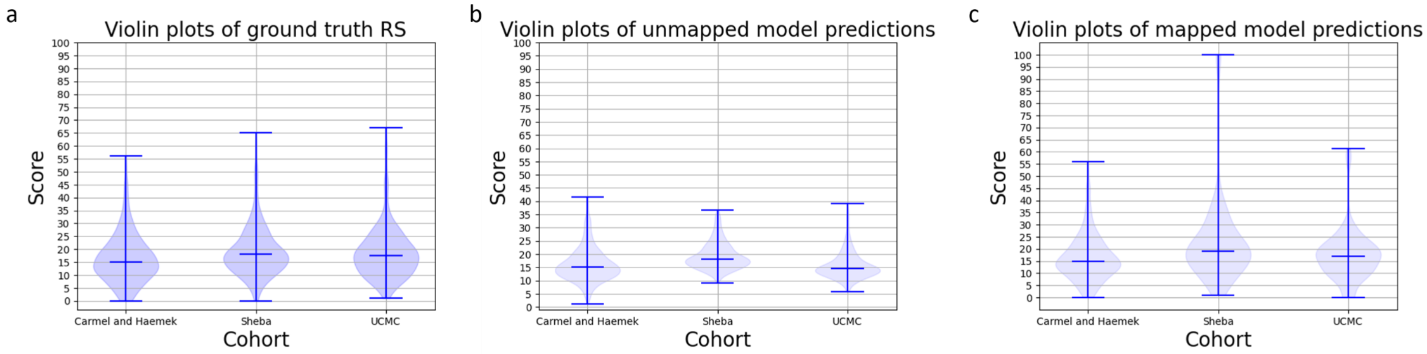

Violin plots of the ground-truth RS ***(a)****, unmapped model predictions* ***(b)****, and the model predictions mapped via histogram matching (see methods)* ***(c)****. Blue horizontal lines represent the minimal, maximal, and median score for each dataset, and grey horizontal lines are added for ease of comparison.*

Checklist for supervised clinical ML study

Before paper submission			
**Study design (Part 1)**	**Completed: page number**		**Notes if not completed**
The clinical problem in which the model will be employed is clearly detailed in the paper.	☑	2,3	
The research question is clearly stated.	☑	2,3	
The characteristics of the cohorts (training and test sets) are detailed in the text.	☑	7,20,22	
The cohorts (training and test sets) are shown to be representative of real-world clinical settings.	☑	7	
The state-of-the-art solution used as a baseline for comparison has been identified and detailed.	☑	2,6	
**Data and optimization (Parts 2, 3)**	**Completed: page number**		**Notes if not completed**
The origin of the data is described and the original format is detailed in the paper.	☑	7	
Transformations of the data before it is applied to the proposed model are described.	☑	7	
The independence between training and test sets has been proven in the paper.	☑	3,8	
Details on the models that were evaluated and the code developed to select the best model are provided.	☑	7,8,10	
Is the input data type structured or unstructured?	☐ Structured ☑ Unstructured		
**Model performance (Part 4)**	**Completed: page number**		**Notes if not completed**
The primary metric selected to evaluate algorithm performance (eg: AUC, F-score, etc) including the justification for selection, has been clearly stated.	☑	8	
The primary metric selected to evaluate the clinical utility of the model (eg PPV, NNT, etc)	☑	8	

including the justification for selection, has been clearly stated.			
The performance comparison between baseline and proposed model is presented with the appropriate statistical significance.	☐		Not relevant
**Model Examination (Parts 5)**	**Completed:**	**page number**	**Notes if not completed**
Examination Technique 1[a]	☑	3,15,16	
Examination Technique 2[a]	☑	4,17	
A discussion of the relevance of the examination results with respect to model/algorithm performance is presented.	☑	6	
A discussion of the feasibility and significance of model interpretability at the case level if examination methods are uninterpretable is presented.	☑	7	
A discussion of the reliability and robustness of the model as the underlying data distribution shifts is included.	☑	8,9	
Common examination approaches based on study type:<br> For studies involving exclusively structured data coefficients and sensitivity analysis are often appropriate<br>* For studies involving unstructured data in the domains of image analysis or NLP: saliency maps (or equivalents) and sensitivity analysis are often appropriate			
**Reproducibility (Part 6): choose appropriate tier of transparency**			**Notes**
Tier 1: complete sharing of the code		☑	
Tier 2: allow a third party to evaluate the code for accuracy/fairness; share the results of this evaluation		☐	
Tier 3: release of a virtual machine (binary) for running the code on new data without sharing its details		☐	
Tier 4: no sharing		☐	

PPV: Positive Predictive Value

NNT: Numbers Needed to Treat

[a] Common examination approaches based on study type: for studies involving exclusively structured data, coefficients and sensitivity analysis are often appropriate; for studies involving unstructured data in the domains of image analysis or natural language processing, saliency maps (or equivalents) and sensitivity analyses are often appropriate. Select 2 from this list or chose an appropriate technique, document each technique used on the appropriate line above.
